# Autoantibodies Which Bind to and Activate Keratinocytes in Systemic Sclerosis

**DOI:** 10.3390/cells12202490

**Published:** 2023-10-20

**Authors:** Carine Moezinia, Valerie Wong, James Watson, Lydia Nagib, Sandra Lopez Garces, Siyu Zhang, Bahja Ahmed Abdi, Florence Newton, David Abraham, Richard Stratton

**Affiliations:** UCL Centre for Rheumatology, Royal Free Hospital, UCL Division of Medicine, London NW3 2QG, UKphingsue_wong@hotmail.com (V.W.); james.watson10@nhs.net (J.W.); lydiagadalla.nagib@nhs.net (L.N.); fnewton@arsenal.co.uk (F.N.);

**Keywords:** scleroderma, autoantibody, epithelial cell, fibrosis

## Abstract

Systemic sclerosis (SSc) is a multisystem connective tissue disease characterised by pathological processes involving autoimmunity, vasculopathy and resultant extensive skin and organ fibrosis. Recent studies have demonstrated activation and aberrant wound healing responses in the epithelial layer of the skin in this disease, implicating the epithelial keratinocytes as a source of pro-fibrotic and inflammatory mediators. In this paper, we investigated the role of Immunoglobulin G (IgG) autoantibodies directed against epithelial cells, as potential initiators and propagators of pathological keratocyte activation and the ensuing SSc fibrotic cascade. A keratinocyte cell-based ELISA is used to evaluate the binding of SSc IgG. SSc skin biopsies were stained by immunofluorescence for the presence of IgG in the keratinocyte layer. Moreover, IgG purified from SSc sera was evaluated for the potential to activate keratinocytes in tissue culture and to induce TLR2 and 3 signalling in reporter cell lines. We demonstrate enhanced binding of SSc IgG to keratinocytes and the activation of these cells leading to the release of IL-1α, representing a potential initiating pathway in this disease.

## 1. Introduction

Scleroderma (systemic sclerosis; SSc) is characterised by a complex and heterogeneous connective tissue disease process that results in fibrosis of the skin and internal organs [1]. Dysregulated adaptive and innate immunity, as well as endothelial damage, are apparent at the earliest stages of disease, whereas persistent myofibroblast activation determines the subsequent skin- and organ-based fibrosis [2]. In essence, SSc represents an abnormally enhanced wound healing process leading to scarring and fibrosis of the skin and internal organs, via extracellular matrix (ECM) deposition. This leads to organ dysfunction and failure, linked to the increased mortality in this group of patients. The primary cause of death in SSc has now shifted from, historically, scleroderma renal crisis to lung fibrosis, pulmonary hypertension and cardiac disease [3].

Whilst the precise cellular and molecular mechanisms are not fully elucidated, several types of autoantibodies have been identified in the sera of SSc patients, including a range of well-established disease-specific antinuclear antibodies such as anti-RNA polymerase (ARAs), anti-centromere (ACAs) and anti-topoisomerase antibodies (ATAs), used clinically to stratify patients [4] and present at the earliest stages [5]. There are also less well-studied and clinically less characterised antibodies defined by binding to and the activation of molecular and cellular targets; including anti-endothelial cell antibodies (AECAs) [6,7], anti-fibroblast [8], anti-matrix metalloproteinase, anti-platelet-derived growth factor receptor (PDGFR) [9] and antifibrillin-1 antibodies [10], each of which may contribute to disease pathogenesis or otherwise represent secondary phenomena. Endothelial cell activation by autoantibodies (AECAs) is considered an important early mechanism in SSc, providing a link between the immune system, vascular damage and fibrosis, through the adhesion and recruitment of immune cells and mobilisation of perivascular stem cells which contribute to the pathogenic tissue myofibroblasts. AECAs are reported in 44–84% of SSc patients and are associated with more severe vascular involvement [11]. Furthermore, SSc autoantibodies may stimulate tissue-resident fibroblasts directly via the Ha-Ras pathways, converting them into activated myofibroblasts, which produce increased levels of collagens and other extracellular matrix (ECM) components characteristically overexpressed by SSc fibroblasts [12].

There is a growing recognition that epithelial cells play a crucial role in the regulation of tissue homeostasis and, when damaged or activated, contribute to the development of a range of fibrotic diseases [13,14]. The pathogenic role of epithelial cells may be highly relevant to SSc given the increasing knowledge of epithelial phenotype changes in this disease [15,16,17,18,19,20]. As well as clinically evident changes in pigmentation [17], the SSc epidermis exhibits an activated wound-like phenotype with delayed keratinocyte terminal differentiation and expression of wound cytokeratins [21]. In addition, in co-culture systems, the epidermis causes the induction of myofibroblasts [15] and exhibits pro-inflammatory effects [18], as well as having a gene expression profile correlating with SSc skin score, identified by single-cell analysis [20]. Moreover, because epithelial cells are covering the outermost layer of the skin, as well as forming the lining cell layer of internal organs such as the respiratory, renal and gastrointestinal tracts, they constitute an integumental cell surface with maximal exposure to environmental factors [22] and collocate with major sites of clinically evident fibrosis in SSc.

Thus, the perturbation of autoantibody affinity to tissue-resident cells, including epithelial cells, may provide a pathogenic link between dysregulated immunity and fibrosis in SSc; however, further mechanistic evidence is needed. In this paper, we investigate and elucidate the role of IgG directed at epithelial cells in SSc patients, as a potential driver of pathological keratinocyte activation and promotor of the SSc fibrotic cascade.

## 2. Materials and Methods

Patients: Patients under the care of the UCL Centre for Rheumatology (Royal Free Hospital) who fulfilled the 2013 ACR/EULAR criteria of diagnosis were included [23]. Written informed consent was obtained from all patients and healthy controls included in the study. Plasma was isolated from blood sampled from sequential consenting patients attending for assessment and then stored at −80 °C prior to assay.

Anti-epithelial cell IgG binding assay: Primary human keratinocyte cells (Invitrogen, San Diego, CA, USA) were cultured with serum-free defined keratinocyte medium (Invitrogen) supplemented with L-glutamine and then sub-cultured in 96-well microtitre plates pre-coated with gelatin. Once the cells were confluent, usually at 48–72 h, the media were removed and the cells were fixed by adding 200 µL of 0.1% glutaraldehyde in PBS to each well, then they were left for 10 min at 4 °C. Cells were then washed gently 4 times in PBS and left to dry overnight. Fixed cells were examined for confluence by microscopy and stored at 4 °C prior to use in the anti-epithelial cell assay.

Non-specific binding protein was blocked with 10% milk powder in PBS. An amount of 200 µL of milk/PBS was added to each well and left at room temperature for 15 min followed by aspiration and replacement of milk/PBS, which was repeated for 4 cycles. Test plasma samples were diluted 1/10, 1/80, 1/320 and 1/1280 in 10% milk powder in PBS. An amount of 50 µL of the test sample was added to each microtitre well and assayed in duplicate for each sample. The plates were left at room temperature for 1 h and then washed 6 times with 10% milk powder PBS. Keratinocyte binding of IgG was detected by 60 min incubation with HRP-conjugated rabbit anti-human IgG (DAKO P0214) diluted 1/500 in milk powder PBS at 50 µL per well, then it was washed 5 times in PBS and the bound horse radish peroxidase activity was assayed by colorimetric analysis of the peroxidation of ortho-phenylenediamine dihydrochloride (OPD) at pH 5, using OPD buffer (12.4 g of Na_2_HPO_4_._12_H_2_0, 350 mL water, pH adjusted to 5). An amount of 100 µL of the substrate solution was added to each microtitre well and the optical density was read at 450 nm with a ELISA plate reader. Plates were read after 15 min.

Following preliminary assays, a dilution of 1/80 was determined as giving optimal differential binding of healthy control (HC) and SSc plasma and was used in the subsequent analysis of HC, limited cutaneous SSc (lcSSc) and diffuse cutaneous SSc (dcSSc) plasma samples (each *n* = 30) (clinical features and therapy shown in Table 1). A strong binding SSc plasma sample was included in each subsequent ELISA assay as positive control and used to determine the binding titre, and each subsequent assayed sample was expressed as percent above the background optical density.

Purification of SSc patients’ IgG using Protein A column: The Pierce/Perbio Protein A column kit (#44667) was used to isolate IgG from SSc and HC plasma samples. The Protein A column was equilibrated with 5 mL of the Immunopure (A) IgG Binding buffer. Each diluted plasma sample was then applied to the column and allowed to flow completely into the gel. The column was then washed with 15 mL of the Immunopure IgG Binding buffer, and then the bound IgG was eluted with 5 mL of the Immunopure IgG Elution buffer. Each 1 mL of elution buffer was collected in collection tubes and assayed by absorbance at 280 nm for the total IgG protein content. The second 1ml fraction was found to contain the highest levels of IgG and was stored at −80 °C and used in subsequent experiments.

Immunohistochemistry of Skin Biopsy Material: Punch biopsies measuring 4 mm were obtained from the involved forearm skin of 6 early-stage dcSSc patients (under 2 years disease duration) and matched sites of 6 HCs. Biopsies were formalin-fixed and then embedded in paraffin prior to sectioning at 5 µm. Standard immunohistochemistry techniques were applied to detect any potential in situ binding of IgG to the epidermal tissue, using rabbit anti-human IgG polyclonal antibody (DAKO #P0214), and secondary fluorescent-labelled goat anti-rabbit IgG and 4′,6-diamidino-2-phenylindole (DAPI) were used to identify nucleated cells.

Cell-viability assay: The potential toxicity of the SSc IgG on human keratinocytes was tested using the tetrazolium salt WST-1 (1644 807; Boehringer Mannheim Biochemicals Inc., Indianapolis, IN, USA). An amount of 10 µL of WST-1 was added per well to cells cultured in 100 µL of culture medium with 0–120 µg/mL SSc IgG, in 96-well plates, incubated for a further 4 h and the colour conversion was measured by reading the absorbance of light at 450 nm with a reference wavelength of 600 nm.

Assay for IL-1α release by keratinocytes: Primary human keratinocytes (Invitrogen) were treated in tissue cultures with SSc and control IgG, purified as above using the Protein-A columns. The normal human keratinocytes (Invitrogen) were passaged two or three times in a 6-well dish containing fully supplemented keratinocyte basic medium (Invitrogen). Once reaching 70% confluency, the cells were maintained, with or without the addition of SSc IgG or HC IgG (0, 10 and 100 μg/mL). Conditioned media, as well as cell lysates, were collected at the end of a 24 h treatment period and assayed for cell layer and secreted media IL-1α levels by an R&D Quantikine Human IL-1α Immunoassay (R&D # DLA50, Minneapolis, MN, USA).

Human epithelial reporter cell lines for TLR 2 and 3: In order to investigate the potential to induce innate responses by the SSc IgG, HEK reporter cell lines (human embryonic tissue cell line) transfected with human TLR2 or TLR3 carrying a reporter gene for NFκB linked to SEAP (HEK-Blue™-hTLR2 Cells and -hTLR3 Cells, Invivogen, CA, USA, #hkb-htlr2 &3) were cultured with or without a range of concentrations of SSc IgG (0–100 µg/mL) for 3 h. For these assays, SSc IgG was purified from plasma samples demonstrating positive binding in the above anti-epithelial cell antibody assays. Samples were included from 14 lcSSc subset and 7 dcSSc subset patients, as well as 6 HC individuals. Media were removed after 3 h of stimulation by IgG and then assayed for the SEAP reporter gene product.

Immunofluorescence to detect uptake of SSc IgG by HaCat cells: In vitro HaCaT cells were grown in DMEM supplemented with 10% FBS and then transferred to 8-well chamber slides (BD Bioscience, Bedford, MA, USA). After serum starvation, the cells were treated with SSc or healthy control IgG at 40 μg/mL (both *n* = 3 replicates), then at timepoints 10 min and 3 h, they were washed with PBS and then fixed in ice-cold methanol–acetone for 5 min at 4 °C. After subsequent PBS washing, non-specific binding was blocked with 10% serum (Vector Labs, Newark, CA, USA) for 30 min. Diluted antibodies to human IgG (Fab-specific) labelled with FITC (Sigma, St. Louis, MO, USA, F5512) or human IgG (Fc-specific) FITC (Sigma F9512) were then applied, and after 30 min, cells were washed in 0.05% PBST. Finally, cells were washed and mounted with Vectashield media (Vector Labs) containing 4′,6-Diamidino-2-Phenylindole (DAPI) and examined with a Zeiss Axioskop Mot Plus microscope using a fluorescence-detecting camera (Carl Zeiss, Gener, Germany).

Statistical analysis: GraphPad Prism software (Graphed Prism 10.0.2) was used throughout. Populations were first compared by ANOVA, followed by nonparametric testing and then corrected for multiple comparisons to determine the significance of treatment effects.

## 3. Results

**Figure 1:**
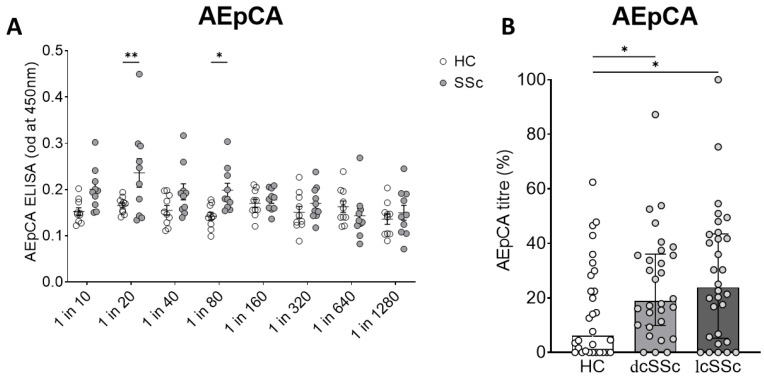
Anti-epithelial cell antibody ELISA in SSc and control plasma. (**A**) A keratinocyte cell-based ELISA was used initially to screen *n* = 10 SSc and healthy control (HC) plasma samples at a range of dilutions, IgG binding was determined by labelled secondary anti-human IgG, absorbance at 450 nm. Optimal differential binding was obtained at a 1 in 80 dilution. (**B**) Subsequently, diffuse cutaneous SSc (dcSSc), limited cutaneous SSc (lcSSc) and healthy control (HC) plasma samples (each *n* = 30) were assayed at a 1 in 80 dilution. In each ELISA plate, a single strong binding positive control sample was included (ascribed 100%) and ELISA titre was determined as percent maximal optical density above the background. * = *p* < 0.05 ** = *p* < 0.01.

Anti-epithelial cell antibody ELISA assay: Initially, we focused on testing whether IgGs from SSc plasma were able to bind fixed keratinocytes cultured in vitro, using the anti-epithelial cell antibody ELISA (AEpCA) as described. An initial screening study was performed to test the performance of the assay using 10 SSc samples and 10 healthy control samples at dilutions from 1/20 to 1/1280 which showed enhanced and dose-dependent binding of the SSc IgG (ANOVA, *p* < 0.001 for overall effect of SSc vs. HC IgG, *p* < 0.0048 for dose effect) (Figure 1A). A dilution of 1/80 was found to give the best performance in the assay with the optimal differential between the binding of SSc and HC IgG and was used in subsequent assays (corrected *p* < 0.027 for SSc vs. HC at 1/80 dilution).

At a dilution of 1 in 80, 30 further HC samples, 30 dcSSc samples and 30 lcSSc samples were screened by anti-epithelial cell ELISA (Figure 1B), demonstrating overall differences between groups (ANOVA *p* < 0.033) and elevated binding in both lcSSc and dcSSc patient samples (Kruskal–Wallis test *p* < 0.014 for lcSSc vs. HC, *p* < 0.041 for dcSSc vs. HC). Although there was a trend for median anti-epithelial cell ELISA values to be higher in lcSSc than in dcSSc subgroups, these effects did not reach significance. Furthermore, clinical and laboratory characteristics were compared between SSc patients with positive AEpCA titre (defined as AEpCA titre above upper 95% CI for binding titre in healthy controls = 22.5%). Amongst those patients with positive AEpCA, there was a trend towards higher skin scores, higher frequency of the ATA antibody profile, more interstitial lung disease and higher use of immunosuppressive treatments (Table 2).

**Figure 2:**
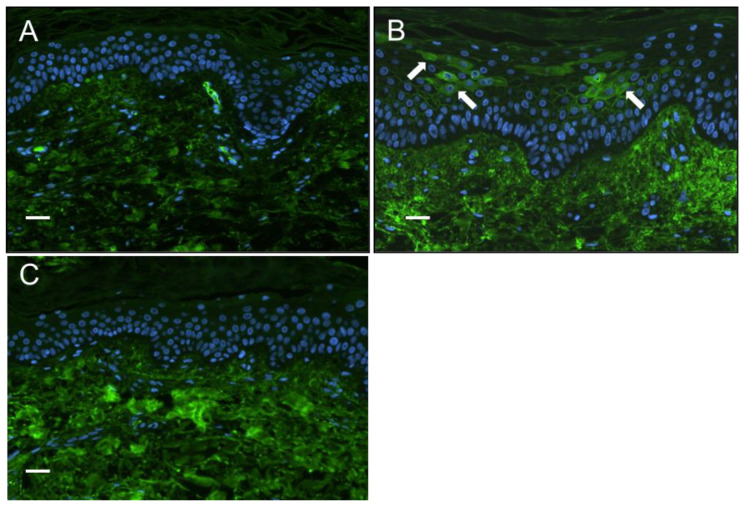
Presence of IgG in the epidermis of systemic sclerosis patients. Immunostaining of SSc and healthy control skin biopsy material for the presence of IgG by indirect immunofluorescence (green). (**A**) Healthy controls’ epidermal cell layers did not stain positive for IgG. (**B**) Some but not all SSc patients (2 out of 6) stained positive for IgG which appeared to stain the cell surface and the cytoplasm of keratinocytes in the granular layer (indicated by arrows). (**C**) No primary IgG control of SSc section, showing no staining of epidermis. Fluorescence of the dermis layer due to autofluorescence of collagen was seen in all sections, with more extensive staining in SSc (scale bar 50 μm).

Positive staining for the presence of IgG in the epidermis of SSc patients’ skin biopsies: Furthermore, to explore the possibility of patient IgG binding to keratinocytes in vivo, we conducted immunostaining of sections of diffuse cutaneous SSc (dcSSc) and healthy skin biopsy material (both *n* = 6) to detect evidence of autoantibody binding in the epidermis. Our results demonstrated the presence of IgG in the cytoplasm and located on the cell surface in some, but not all, SSc epidermal sections (observed in two out of six patients), whereas no healthy control sections showed positive staining (Figure 2).

**Figure 3:**
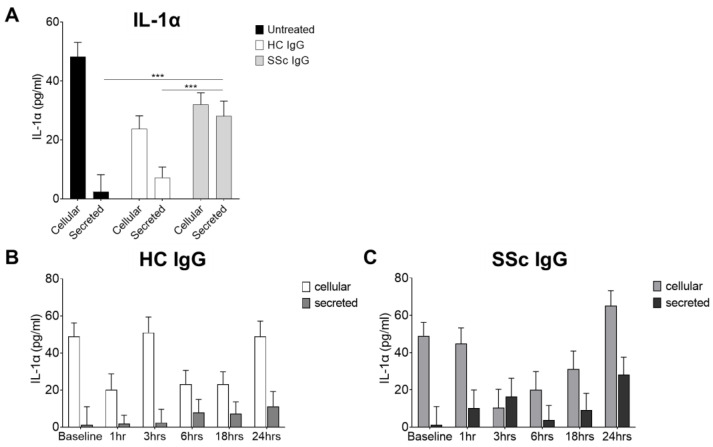
Systemic sclerosis IgG promotes release of IL-1α by human keratinocytes. (**A**) Normal human keratinocytes cultured in 6-well plates were treated with IgG (10 μg/mL) from SSc patients and controls. After 24 h, IL-1α was assayed in media and cell layers by ELISA. Treatment with SSc IgG at 10 μg/mL lead to a significant increase in the release of IL-1α, not seen with control IgG treatment or media only. (**B**) A time course of IL-1α release following treatment with control IgG and (**C**) SSc IgG (10 µg/mL) was determined by treating keratinocytes in 6-well plates, removing media and lysing the cell layer at various timepoints. Treatment with SSc IgG but not HC trended to a rapidly evoked and biphasic induction of IL-1α release (p NS). *** = *p* < 0.001.

Activation of keratinocytes by systemic sclerosis IgG: Because of the enhanced binding activity of SSc sera in AEpCA ELISA in vitro, it was hypothesised that SSc immunoglobulins could be binding to and activating keratinocytes in the disease tissue. IgG purified on protein A columns from severe dcSSc patients and from healthy controls was assayed for the capacity to activate keratinocytes in vitro. Isolated IgG from three patients with recent onset diffuse SSc which tested with high titre in AEpCA ELISA and from three healthy control sera were used to activate cultured primary human keratinocytes. IL-1α levels in the conditioned media versus the keratinocyte cell layer levels were used as a readout of keratinocyte activation. Treatment with SSc IgG (10 μg/mL) was associated with significantly higher levels of IL-1α secreted into the media, when compared to control IgG treatment or media alone (*p* < 0.0001 for SSc IgG treated versus untreated, *p* < 0.002 for SSc IgG versus HC IgG) (Figure 3A). In other experiments, a time course was determined by treating keratinocytes with the same SSc and HC IgG (10 μg/mL) and removing media and the cell layer at various timepoints for assay. Treatment with SSc IgG but not HC IgG resulted in a rapid induction of IL-1α release, apparent by 3 h with SSc IgG, although this did not reach significance (Figure 3B,C). No significant cytotoxicity was seen with concentrations of SSc IgG of 10–120 µg/mL in the MTT cyto-toxicity assay.

**Figure 4:**
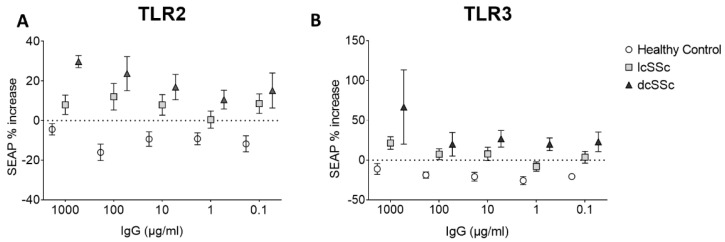
Effect of SSc patients’ plasma IgG on TLR2 and TLR3 expressing reporter cell lines. HEK reporter cell lines overexpressing (**A**) TLR2 or (**B**) TLR3 were stimulated with HC and SSc IgG for 3 h after which media were removed and assayed for SEAP as a marker of reporter gene expression indicating activation of the TLR pathway (*n* = 6 HC, 14 lcSSc and 7 dcSSc plasma samples analysed).

Effect of SSc IgG on TLR 2 and 3 signalling pathways in HEK cells: Because of the induction of innate responses, an attempt was made to measure the effect of SSc and HC IgG on the TLR pathway, an important innate immune pathway in epithelial cells, using HEK hTLR2 and hTLR3 reporter cell lines. Treatment with HC IgG led to a small decrease in reporter gene expression in both cell types, whereas lcSSc and dcSSc IgG induced SEAP expression in both TLR2 and TLR3 cells (TLR2 cells, *p* < 0.0001 for overall difference between groups, *p* < 0.012 for effect of lcSSc vs. HC IgG, *p* < 0.0023 for dcSSc vs. HC IgG, higher levels in dcSSc vs. lcSSc IgG, *p* < 0.014, ANOVA followed by Tukey’s test corrected for multiple comparisons) (TLR3 cells *p* < 0.0003 for overall difference between treatments, *p* < 0.025 for effect of lcSSc vs. HC IgG, *p* < 0.0002 for dcSSc vs. HC IgG, higher in dcSSc vs. lcSSc IgG, *p* < 0.032, ANOVA followed by Tukey’s test corrected for multiple comparisons) (Figure 4). These results indicated an effect of SSc IgG on innate signal induction in these cells and a more profound response in dcSSc compared with lcSSc.

**Figure 5:**
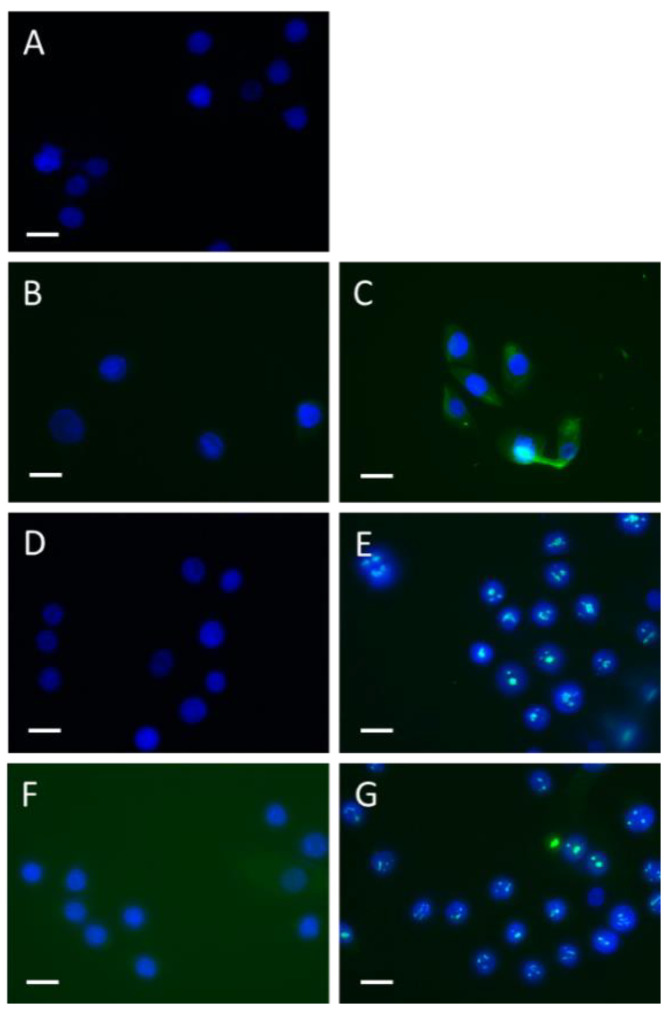
Time course and apparent nuclear translocation of SSc IgG in HaCat cells. HaCat epithelial cells were cultured with or without addition of SSc or HC IgG (40 μg/mL) and then washed and fixed at various timepoints and stained for human IgG. (**A**) Shows no primary antibody IgG control, (**B**) HC IgG Fc at 10 min, (**C**) SSc IgG Fc at 10 min, (**D**) HC Fc at 3 h, (**E**) SSc IgG Fc at 3 h, (**F**) HC Fab at 3 h, (**G**) SSc IgG Fab at 3 h (bar = 20 μm).

Uptake of SSc but not HC IgG by keratinocytes: To determine whether SSc IgG was capable of entering keratinocytes, HaCat cells were cultured with or without the addition of SSc IgG or HC IgG. The cells were then fixed at two different timepoints, 10 min and 3 h, and subject to immunostaining using both Fc-specific and Fab-specific antibodies for human IgG. The results demonstrated that at 10 min, SSc IgG staining was found, indicating that the antibody had translocated to the cytoplasmic compartment, whereas HC IgG did not exhibit any staining. Additionally, after 3 h, SSc IgG was found to stain within the nucleus, strongly suggesting that the IgG had translocated to the nuclear compartment (Figure 5) (replicate numbers too low for statistical inference).

## 4. Discussion

In this study, we clearly show that IgG from dcSSc and lcSSc patients recognises components of epithelial cells and exhibits significantly elevated binding compared with control IgG. We demonstrate positive binding of SSc patients’ IgG to fixed human keratinocytes, which did not differ significantly between lcSSc and dcSSc patient groups. It has been previously established that the epidermis in SSc patients undergoes critical changes, including a proposed model of TGF-β-induced activation of epithelial cells, causing an epithelial-to-mesenchymal transition (EMT)-like phenomena and resulting in the hallmark damaging and pathogenic fibrosis of the disease [19]. Reflecting on the potential for autoantibodies to initiate cellular damage in various other autoimmune diseases and due to the current gap in knowledge of the role of anti-epithelial cell antibodies specifically in SSc, we explored whether IgG antibodies targeting epithelial cells in skin lesions of SSc patients could be the potential driver of pathological keratocyte activation and the initiation of the fibrotic cascade.

Of note, clinical differences were observed in SSc patients testing positive for AEpCA versus AEpCA-negative patients, including a trend for interstitial lung disease, higher skin score and the presence of the ATA antibody profile. These changes would be consistent with a more severe phenotype in the AEpCA-positive group supported by the observed more frequent need to provide immunomodulatory treatment. It is also plausible, due to the observed shared function and identity between different epithelial populations [24], that the AEpCA could target pulmonary and intestinal epithelial cells, in addition to keratinocytes in such patients, or that more severe skin activation is promoting the characteristic systemic aspects of the disease.

As a limitation, we have not defined the target antigen(s) involved, which has also been a limitation for other studies of pathogenic antibodies in the disease; for example, the endothelial cell antibodies in the disease and even specific anti-PDGFR antibodies [9] have not been a consistent finding between studies. Moreover, in our study, we found that SSc patients’ IgG activated the keratinocytes, inducing the release of IL-1α, a similar effect to that shown previously for endothelial cells [6]. Furthermore, SSc IgGs were found to induce signalling in TLR2 and TLR3 reporter cells, representing a potential mechanism, because these receptors are expressed by epithelial cells.

Some of these findings are consistent with previous studies from Rubin demonstrating that SSc IgG induces innate responses via uptake into the endosomal compartment [25]. Also of interest, in our study, healthy control IgGs were found to dimmish the TLR signalling in reporter cells, indicating a potential immunoregulatory effect of healthy control IgG.

The main early pathophysiological mechanisms in SSc are believed to include both microvascular damage as well as the presence of autoantibodies. It is intriguing to suggest that the IgG activation of epithelial cells could be included amongst the earliest disease-initiating mechanisms. Our results indicate variation in the extent of these effects, which is entirely consistent with the known heterogeneous nature of SSc as a clinical entity and the need to stratify patients. Where environmental factors have been implicated such as in solvent exposures [26], these might be of relevance to initiating epithelial mechanisms, whereas endothelial cell damage could be triggered by systemic factors such as viral infection [27] so that both external integumental activation and internal endothelial activation could operate as dual initiating factors. These mechanisms may initiate inflammatory cascades and trigger the transition of normal fibroblasts to myofibroblasts, responsible for abnormal extracellular matrix deposition in affected tissues and eventual organ damage and deterioration.

Although our results demonstrate that anti-epithelial cell antibodies are present in SSc patients’ plasma, our study has several limitations, including a small sample size and the lack of a clear target antigen involved in the binding. However, the triggering of innate immune mechanisms in the epithelial cell layer by IgG is of significant interest, as a potential mechanism that could be addressed by therapeutic antagonists, especially if the precise molecular mechanisms can be better elucidated.

## 5. Conclusions

In conclusion, in this study, we have shown evidence of anti-epithelial cell antibodies in the plasma of SSc patients and demonstrated that SSc IgGs are capable of activating and triggering relevant innate immune mechanisms in epithelial cells. This represents a potential mechanism triggering epithelial activation and contributing to downstream fibrosis in the skin, as well as a potential target for the future discovery of new and effective therapies.

## Figures and Tables

**Table 1:** Clinical and laboratory characteristics of SSc patients included in the anti-epithelial cell antibody ELISA profiling. ATA = anti-topoisomerase, ARA = anti-RNA polymerase, ACA = anti-centromere, AFA = anti-fibrillarin, ANA = antinuclear antibody without disease-specific antibody, PM/Scl = polymyositis/scleroderma, ILD = interstitial lung disease, SRC = scleroderma renal crisis, PAH = pulmonary arterial hypertension, Pred = prednisolone, MMF = mycophenolate mofetil, MTX = methotrexate, Cyclo = cyclophosphamide, HCQ = hydroxychloroquine, Aza = azathioprine.

	Diffuse Cutaneous SSc (*n* = 30)	Limited Cutaneous SSc (*n* = 30)
Gender	M 4, F 26	M 3, F 27
Autoantibody subset	ATA 11, ARA 10, ACA 2, ANA 4, AFA 2, PM/Scl 1	ATA 8, ACA 18, ANA 3, Neg 1
Organ involvement	ILD 12, SRC 2, PAH 4	ILD 8, SRC 1, PAH 4
Skin score (mRSS, Median, Range)	16 (4–40)	4 (0–12)
Disease duration (years, Median, Range)	7.4 (2.8–27)	8 (0.6–21.8)
Immunomodulatory therapy	15/30 (Pred 10, MMF 7, MTX 3, Cyclo 1, HCQ 1, Aza 1)	7/30 (Pred 5, MMF 2, HCQ 2, Aza 1)

**Table 2:** Clinical and laboratory characteristics in SSc patients positive or negative for AEpCA. Trends towards higher rates of ATA antibody, presence of ILD and need for immunomodulatory treatment were seen in AEpCA-positive patients. ATA = anti-topoisomerase, ARA = anti-RNA polymerase, ACA = anti-centromere, AFA = anti-fibrillarin, ANA = antinuclear antibody without disease-specific antibody, PM/Scl = polymyositis/scleroderma, ILD = interstitial lung disease, SRC = scleroderma renal crisis, PAH = pulmonary arterial hypertension, Pred = prednisolone, MMF = mycophenolate mofetil, MTX = methotrexate, Cyclo = cyclophosphamide, HCQ = hydroxychloroquine, Aza = azathioprine. * chi square analysis. ** Mann–Whitney test. NS = not significant at *p* < 0.05.

Characteristic	AEpCA Negative (*n* = 30)	AEpCA Positive (*n* = 30)	*p* Value
Diffuse vs. Limited subset	15 D: 15 L	15 D: 15 L	NS
Gender	2 M, 28 F	5 M, 25 F	NS
Autoantibody subset	6 ATA, 7 ARA, 12 ACA, 3 ANA, 1 AFA, 1 Neg	13 ATA, 3 ARA, 8 ACA, 4 ANA, 1 PM/Scl, 1 AFA	*p* = 0.052 for ATA *
Organ involvement	7 ILD, 2 SRC, 4 PAH	13 ILD, 1 SRC, 4 PAH	NS
Skin score (mRSS 0–51, Median, Range)	5 (0–24)	9 (0–40)	*p* = 0.031 **
Disease duration (years, Median, Range)	7.7 (2.5–27)	8 (0.6–19)	NS
Immunomodulatory therapy	10/30 (6 Pred, 5 MMF, 2 MTX, 1 HCQ)	12/30 (9 Pred, 4 MMF, 1 MTX, 1 Cyclo, 2 Aza, 2 HCQ)	NS

## Data Availability

In general each individual data point has been presented in the dot plot charts. The data presented in this study are available on request from the author for correspondence.
